# Separation, Characterization and Dose-Effect Relationship of the PPARγ-Activating Bio-Active Constituents in the Chinese Herb Formulation ‘San-Ao Decoction’

**DOI:** 10.3390/molecules14103942

**Published:** 2009-10-09

**Authors:** Ling Zhou, Yu-Ping Tang, Lu Gao, Xin-Sheng Fan, Chun-Mei Liu, De-Kang Wu

**Affiliations:** 1 Jiangsu Key Laboratory for TCM Formulae Research, Nanjing University of Chinese Medicine, Nanjing 210046, China; 2 Nanjing Medical University, Nanjing 210029, China

**Keywords:** San-ao decoction, PPARγ, does-effect, UPLC, quatropde time-of-flight mass spectrometry

## Abstract

San-ao decoction (SAD), comprising Herba Ephedrae, Radix et Rhizoma Glycyrrhizae and Seneb Armeniacae Amarum, is one of the most popular traditional Chinese medicine (TCM) formulae for asthma. Peroxisome proliferator-activated receptors (PPARs) are key regulators of lipid and glucose metabolism and have become important therapeutic targets for various deseases, PPARγ activation might exhibit anti-inflammatory properties in different chronic inflammatory processes. The EtOAc fraction of SAD showed a significant effect on PPARγ activation. A simple and rapid method has been established for separation and characterization of the main compounds in the PPARγ-activating fraction of SAD by ultra-fast HPLC coupled with quadropole time-of-flight mass spectrometry (UPLC-Q-TOF/MS). A total of 10 compounds were identified in the activating fraction of SAD, including amygdalin (**1**), liquiritin (**2**), 6′-acetyliquiritin (**3**), liquiritigenin (**4**), isoliquiritigenin (**5**), formononetin (**6**), licoisoflavanone (**7**), glycycoumarin (**8**), glycyrol (**9**) and quercetin (**10**). The results also characterized formononetin as a predominant component in this fraction. The dose-effect relationship comparison study of formononetin and the EtOAc fraction of SAD by adding formononetin was performed, the results suggested that formononetin was the major component of the EtOAc fraction of SAD responsible for activating PPARγ, and the method will possibly be applied to study the complex biological active constituents of other TCMs.

## 1. Introduction

Traditional Chinese medicines (TCMs) are natural therapeutic remedies used under the guidance of traditional Chinese medical philosophy and the Chinese community worldwide for thousands of years. Most of the TCMs are multi-ingredient formulae, and it is widely accepted that multiple constituents are responsible for their bio-activities [1]. However, due to the complexity of the chemical compositions of TCMs, the bioactive compounds and the therapeutic mechanisms of most TCMs are still unknown. Therefore, it is important and urgent to carry out chemical and pharmacological studies on TCMs.

San-ao decoction (SAD) is a well-known Chinese formula that has been used clinically to treat upper respiratory diseases such as colds and bronchitis for thousands of years. Nowadays, it is widely used in clinical practice for treating bronchitis caused by bacterial and viral infection. SAD decoction consists of three traditional Chinese medicines: Herba Ephedrae, Semen Armeniacae amarum, and Radix Glycyrrhizae, which are all recorded in the Chinese Pharmacopoeia [2].

The use of liquid chromatography-mass spectroscopy (LC/MS) is now commonplace, as the desire for significantly reduced analysis times with increased sample throughput, sensitivity, and resolution has resulted in the research of simultaneous determination on multiple compounds, such as the chemical research of formulae of TCMs [3,4]. Because of the high speed of analysis, sensitivity and confirmation of structural information, ultra-fast high performance liquid chromatograph (UPLC) system coupled with mass spectrometry has become the preferred analytical technique for herbal medicines [5,6,7,8]. Quatropole time-of-flight (Q-TOF) MS shows its unique advantages in providing high selectivity with narrow mass windows over nominal mass chromatograms, accurate mass measurements for elemental composition and structural information.

Peroxisome proliferator-activated receptors (PPARs), which were divided into three subtypes including α, δ and γ, are ligand-activated transcription factors of a nuclear receptor superfamily [9,10], PPARγ is thought to be involved in cell proliferation or carcinogenesis if it is ubiquitously expressed [11,12], and it is also known as potent agents in the treatment of type 2 diabetes, inflammatory diseases, and cancers [13,14]. PPARγ belongs to the group of nuclear hormone receptors consisting of a ligand and DNA binding domain which, upon activation by their respective ligands, bind to specific PPAR response elements (PPREs) in the promoter of their target genes, thus regulating their expression. PPARγ can be activated by naturally occurring ligands. PPARγ is also a nuclear transcription factor originally described as a major regulator in glucose homeostasis and adipogenesis [10], but recent work has shown that PPARγ activation might exhibit anti-inflammatory properties in different chronic inflammatory processes [15]. Previous work has shown inhibition of NF-κB activation by PPARγ activators in various cell types such as endothelial cells and monocyte/macrophages [16,17].

In order to investigate the bio-active components of SAD, the activity of four fractions from SAD decoction on restoring glycemic balance induced by PPARγ was investigated. The results showed the EtOAc fraction had obvious biological activity. Then, a UPLC-Q-TOF/MS method was selected and developed for characterization of constituents in the active fraction of SAD for the first time, and the dose-effect relationship of the main bio-active constituent was also investigated in order to clarify the total bio-active contribution from the constituents.

## 2. Results and Discussion

### 2.1. Effect of the different fraction from SAD on PPARγ

**Figure 1:**
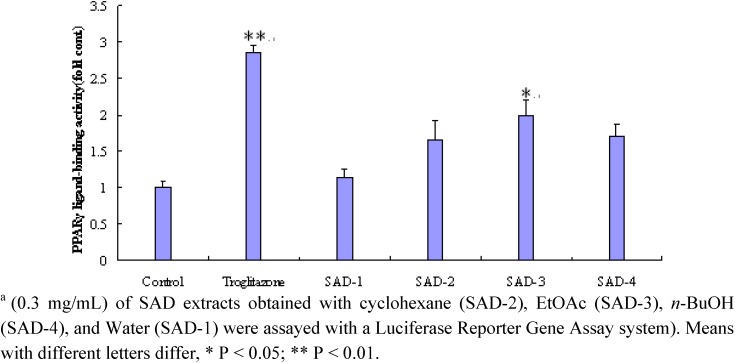
Peroxisome proliferator-activated receptor PPARγ ligand-binding activity of SAD. ^a^

The activities of nonaqueous fractions of SAD with cyclohexane, *n*-butyl alcohol extracted were similar, the ethyl acetate fraction showed obvious activity (Figure 1). Yields of these extracts obtained from cyclohexane, EtOAc, *n*-BuOH and water participation were 4.6, 1.3 and 4.8 g/100 g, respectively. The activities at 0.3 g/mL of the nonaqueous extracts of SAD were equivalent to 40 % of that at 0.5 μmol/L of T174, a synthetic potent PPARγ agonist.

### 2.2. UPLC-Q-TOF/MS analysis of the fraction activiting of PPARγ in SAD

**Figure 2:**
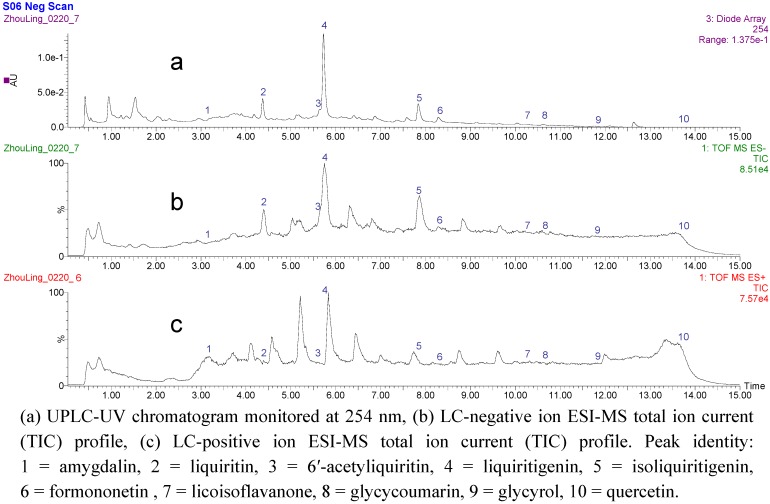
UPLC-Q-TOF/MS/MS analysis of the SAD EtOAc fraction.

**Figure 3:**
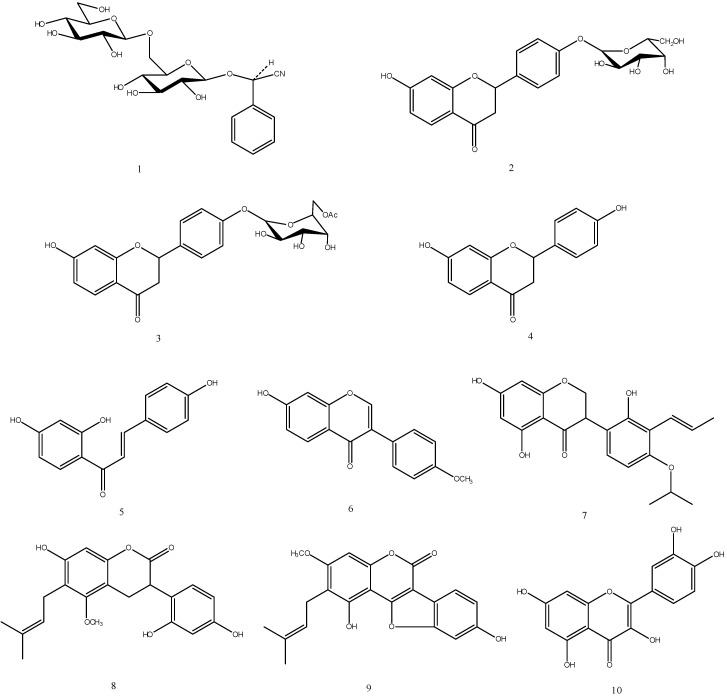
Chemical structures of the compounds identified in the SAD EtOAc fraction.

A UPLC-ESI-MS/MS method was developed to separate and characterize the chemical constituents of the PPARγ-activating EtOAc fraction. Ten identified peaks were separated under the UPLC condition with UV detection, and the chromatography of MS total ion current (TIC) in negative mode was shown in Figure 2. The retention time, m/z value and UV maximum absorption wavelength of each peak was summarized in Table 1. Ten peaks were identified as amygdalin (**1**), liquiritin (**2**), 6′-acetyliquiritin (**3**), liquiritigenin (**4**), isoliquiritigenin (**5**), formononetin (**6**), licoisoflavanone (**7**), glycycoumarin (**8**), glycyrol (**9**) and quercetin (**10**) by comparing the retention time, UV and ESI-MS spectra with authentic standards (Table 1, Figure 3). Chemical structures of the identified compounds are shown in Figure 3 and their MS/MS fragmentations are also shown in Table 1.

**Table 1:** UPLC-Q-TOF/MS analysis of EtOAc fraction from SAD.

Peak no.	Retention time (min)	MS^2^ of fragment Ions	ESI-mass spectra[M-H]^-^	Maximum absorption wavelength λ_max_ (nm)	Identification
1	3.59	385.14, 340.18, 237.08, 187.09, 165.05	456.15	210, 252, 263, 269	amygdalin
2	4.39	417.11, 366.11, 255.06, 151.04, 135.00	418.15	241, 275, 327	liquiritin
3	5.73	417.11, 255.06, 135.00	469.13	230, 258, 277, 320	6′-acetyliquiritin
4	5.75	135.01	255.07	236, 272, 315	liquiritigenin
5	7.68	135.01	255.06	240, 330, 395	isoliquiritigenin
6	8.29	255.06, 135.01	267.06	248, 300	formononetin
7	10.17	297.20, 269.08	353.10	227, 260, 330	licoisoflavanone
8	10.59	353.11, 329.22, 183.02, 141.02	367.11	236, 327, 370, 384	glycycoumarin
9	11.79	335.10, 267.04	365.10	229, 286, 351, 382	glycyrol
10	13.57	227.16	301.16	254, 312	quercetin

### 2.3. Effect of ten compounds from EtOAc fraction on PPARγ

**Figure 4:**
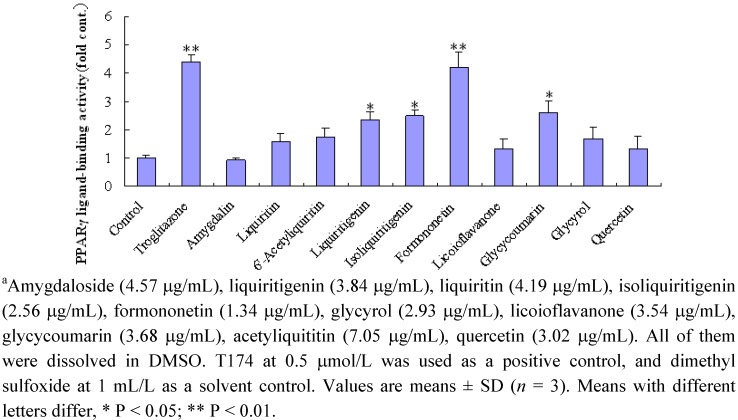
Peroxisome proliferator-activated receptor PPARγ ligand-binding activity of ten identified compounds from the EtOAc of SAD. ^a^

Furthermore, the activities of ten compounds (five concentrations for each compound) from the EtOAc fraction of SAD were evaluated, and the results (the optimum concentration for each compound) are shown in Figure 4. Liquiritin, isoliquiritigenin, glycycoumarin and formononetin (5 μm) showed significant activity.

### 2.4. Dose-effect relationship of formononetin in EtOAc fraction of SAD

**Figure 5:**
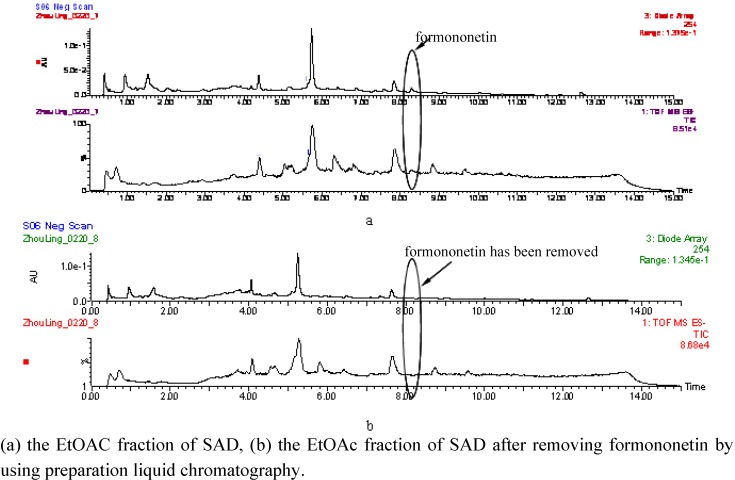
UPLC-Q-TOF/MS/MS analysis of the EtOAc fraction of SAD before and after removing formononetin.

Formononetin was completely removed from the EtOAc fraction of SAD using preparative HPLC; the results are shown in Figure 5. The dose-effect of formononetin is shown in 6, the optimum concentration of formononetin was 1.34 μg/mL. When we gradually added fomononetin into the EtOAc fraction from which the fomononetin had already been removed the results showed almost the same trend as the dose-effect of formononetin. When the concentration of formononetin in the EtOAc fraction was up to 1.34 μg/mL, the activation effect on PPARγ was even better than that of formononetin itself., and the activity of the EtOAc fraction (formononetin was 0.76 μg/mL) was also near that of formononetin (0.76 μg/mL). These results suggested that fomononetin was possibly the major active component for the activation on PPARγ in the SAD EtOAc fraction.

**Figure 6:**
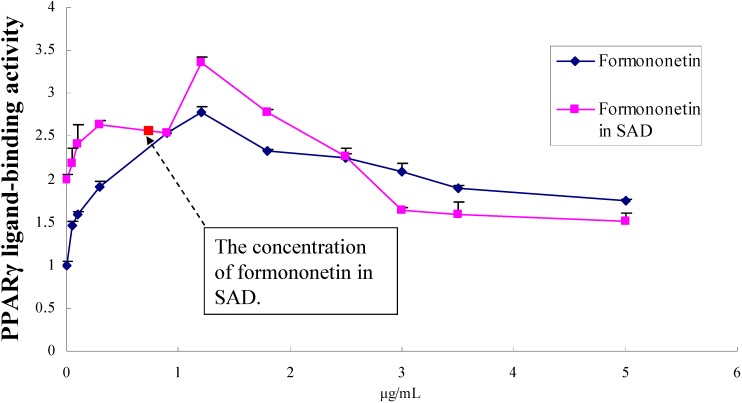
Dose-effect relationship of formononetin in the EtOAc fraction of SAD..

## 3. Experimental

### 3.1. Chemicals and reagents

HPLC grade methanol (Tedia, U.S.A.) was used for HPLC analysis. Reagent grade ethanol (Hanbang Company, Huaian, China) was used for extraction and separation. Amygdalin, liquiritin, 6′-acetyliquiritin, licoisoflavanone, glycycoumarin, glycyrol, quercetin were purchased from the National Institute for the Control of Pharmaceutical and Biological Products (Beijing, China). Liquiritigenin, isoliquiritigenin and formononetin were isolated in our laboratories.

### 3.2. Plant material

Herba Ephedrae (*Ephedra sinica* Stapf), Semen Armeniacae amarum (*Prunus armwniaca* L.var. *ansu* Maxim.) and Radix Glycyrrhizae (*Glycyrrhiza uralensis* Fisch.) were collected from Shanxi, Hebei and Inner Mongolia, respectively. The crude herbs were identified by Dr. Wei Yue of the Nanjing University of Chinese Medicine.

### 3.3. Sample preparation

The sliced crude drug materials (900 g) were extracted twice with boiling water for 2 h each time. After removal of the solvent under reduced pressure to give the water extract, the residue was partitioned sequentially with cyclohexane, EtOAc and *n*-BuOH, respectively, to afford three fractions. Sample for UPLC/MS analysis: EtOAc fraction (100 mg) was dissolved in MeOH (10 mL). 1 μL of the solution was injected into the UPLC/MS analysis. Sample for isolation by preparation liquid chromatography: EtOAc fraction (5 g) was dissolved in MeOH (50 mL). All the solutions were injected into the chromatograph using 500 μL each time.

### 3.4. Equipment and chromatographic conditions

A Waters UPLC ACQUITY^TM^ (Waters Company, U.S.A.) equipped with the MassLynx V4.1 software (Waters) and comprised of a quaternary solvent delivery pump, an online vacuum degasser, an autosampler, a thermostated compartment and a diode array detector, were used for the chromatographic analysis. All separations were carried out on an ACQUITY UPLC BEH C_18_ column (2.1×100 mm, 1.7 μm, Waters). Mobile phase: A = water, and B = methanol. The elution was performed using a linear gradient of 2-10% B within 2 min, 10-30% B within 2-3 min, 30-80% B within 3-13 min, and 80-2% B at the last 2 min. The flow-rate was 0.3 mL min^-1^. The temperature of column was 30 °C. The UV spectrum was recorded from 190 to 400 nm, and injection volume was 1 μL.

### 3.5. Mass spectrometric conditions

Waters Synapt Q-TOF-MS (Waters Company, USA) was connected to the Waters UPLC instrument via an ESI interface for UPLC-MS/MS analysis. Ultra high purity helium was used as the collision gas and high-purity nitrogen (N_2_) as the makeup gas. The optimized parameters in the negative and positive ion mode were as follows: capillary, 3,000.0 V, sample cone, 35.0 V, desolvation temp, 200.0 °C, source temp, 100.0 °C, cone, 58 L·hr^-1^, desolvation 500 L·hr^-1^, collision energy, 35.0 (MS/MS), 5.0 (MS). For full scan MS analysis, the spectra were recorded in the range of m/z 100-1,000.

### 3.6. Equipment and conditions of preparative liquid chromatography

Preparative analysis was carried out on a Waters 2545 Binary Gradient Module with a Waters 2489 UV/Visible Detector, and Waters 2767 Sample Manager (Waters). Separation was achieved by using a SunFire Prep C_18_ OBD column (30 mm × 150 mm, 5 μm, Waters) with a mobile phase of methanol-aqueous phase (containing 0.1% TFA) at a flow rate of 30 mL/min. The percentage of methanol in the mobile phase was programmed as follows: 60% (0 min) - 75% (10 min) - 100% (11 min) - 60% (12 min). The elution was carried out at ambient temperature. The injection volume was 800 μL.

### 3.7. Cell culture and full-length reporter-gene bioassays

Thiazolidinedione (T174), a specific ligand for PPARγ, was kindly provided by Tanabe Seiyaku (Osaka, Japan). It was diluted with dimethyl sulfoxide (DMSO) to prepare the stock solutions (10-100 mM). PPAR ligand-binding activity at 0.5 μmol/L of troglitazone was approximately three-fold of that of the solvent control. CHO, a Chinese hamster ovary cell line (from ATCC), was maintained on 24-well tissue culture plates containing the maintenance medium at 37 °C, in 5% CO_2_. At 80% confluent, 1 μg/well of human PPARγ expression vector, pGl3-3xPPRE-luc plasmid, or control vector respectively was transfected into cells using a Lipofectamine Plus system (Invitrogen). As the negative control, human PPARγ expression vector and pGl3-3xPPRE-luc plasmid were transfected into the cell line. After transfection, the cells were cultured for 24 h, and compounds for ligand assay were added into the medium at appropriate concentrations. After additional 24 h incubation, the cells were lysed for luciferase assay performed using a Luciferase Reporter Gene Assay system (Promega) according to the manufacturer’s protocol.

## 4. Conclusions

A UPLC-Q-TOF/mass method had been established for the first time for the separation and characterization of the main compounds in the SAD fraction activating PPARγ. A total of 10 compounds were identified in the activating SAD fraction, including isoliquiritigenin, formononetin and other flavonoids. The method could rapidly separate and elucidate complex constituents in TCMs, in comparison to other analysis method such as TLC and HPLC. The effects of the compounds and the EtOAc fraction of SAD for activating PPARγ were evaluated by the luciferase activity assay, and the results indicated that some isoflavones, especially formononetin, showed significant activity. Formononetin, a isoflavonoid found in high concentrations in leguminous plant, has been shown to reduce production of proinflammatory mediators in LPS-stimulated macrophages, fibroblasts, and intestinal epithelial cells. It was isolated from *Pueraria thomsonii* as the PPAR-activating compound [18]. The dose-effect relationship comparison study of formononetin and the EtOAc fraction of SAD by adding formononetin was performed, and the results suggested that fomononetin was the major component of the EtOAc fraction of SAD responsible for activating PPARγ. The method could possibly be applied to study complex biological active constituents of other TCMs.
